# A new chigger species of the genus *Hannemania* Oudemans, 1911 (Acariformes: Prostigmata: Leeuwenhoekiidae) parasite of the Miranda’s white-lipped frog *Leptodactylus macrosternum* (Anura: Leptodactylidae) in the Caatinga biome, Brazil

**DOI:** 10.1093/jme/tjaf117

**Published:** 2025-09-24

**Authors:** Gabriela Felix-Nascimento, Ricardo Paredes-León, Ellen Candida Ataide-Gomes, Fabiano Matos Vieira, Geraldo Jorge Barbosa de Moura, Leonardo Barros Ribeiro, Jaqueline Bianque de Oliveira

**Affiliations:** Laboratório de Anatomia dos Animais Domésticos e Silvestres, Universidade Federal do Vale do São Francisco (UNIVASF), Petrolina, Pernambuco, Brazil; Programa de Pós-Graduação em Biociência Animal (PPGBA), Universidade Federal Rural de Pernambuco (UFRPE), Recife, Pernambuco, Brazil; Colección Nacional de Ácaros, Departamento de Zoología, Instituto de Biología, Universidad Nacional Autónoma de México (UNAM), Coyoacán, Ciudad de México, México; Centro de Conservação e Manejo de Fauna da Caatinga (CEMAFAUNA), Universidade Federal do Vale do São Francisco (UNIVASF), Petrolina, Pernambuco, Brazil; Laboratório de Microscopia e Lupas, Universidade Federal do Vale do São Francisco (UNIVASF), Petrolina, Pernambuco, Brazil; Programa de Pós-Graduação em Biociência Animal (PPGBA), Universidade Federal Rural de Pernambuco (UFRPE), Recife, Pernambuco, Brazil; Laboratório de Estudos Herpetológicos e Paleoherpetológicos (LEHP), Universidade Federal Rural de Pernambuco (UFRPE), Recife, Pernambuco, Brazil; Laboratório de Fisiologia Animal, Universidade Federal do Vale do São Francisco (UNIVASF), Petrolina, Pernambuco, Brazil; Laboratório de Parasitologia (LAPAR), Universidade Federal Rural de Pernambuco (UFRPE), Recife, Pernambuco, Brazil

**Keywords:** Trombidiformes, Trombiculoidea, chigger mites, parasites of amphibia, South America

## Abstract

A new chigger species of the genus *Hannemania* Oudemans, 1911 is described, which parasitizes the Miranda’s white-lipped frog, *Leptodactylus macrosternum* Miranda-Ribeiro, 1926 (Anura: Leptodactylidae) in the Caatinga. The new species differs from all other *Hannemania* by having the odontus with more prongs (four) than the other species, except for *H. argentina* Lahille, 1927 that has one to five prongs. However, the new species can be separated from the latter by its fPp and the shape of *bs* seta. This new species is the 29th of the genus and the seventh species of *Hannemania* recorded in Brazil.

## Introduction

The genus *Hannemania* Oudemans, 1911 (Acariformes: Prostigmata: Trombiculoidea: Leeuwenhoekiidae) currently includes 28 species of mites, whose larvae (chiggers) are specific parasites of amphibians (Amphibia: Anura and Caudata) mostly distributed in North and South America (27 species) and only one species in New Caledonia (Oudemans 1917, Sambon 1928, Fuller 1952, Wharton and Fuller 1952, Gould 1956, Loomis 1956, Hoffmann 1990, Wohltmann et al. 2006, Silva-de la Fuente et al. 2016, Mendoza-Roldan et al. 2020, Bassini-Silva et al. 2021). These mites penetrate in the host skin and become completely embedded in it up to six months (Hyland 1961, Anthony et al. 1994).

In Brazil six species of the genus *Hannemania* have been recorded: *H. hylodeus* (Oudemans 1910), *H. newsteadi*  Sambon 1928, *H. stephensis*  Sambon 1928, *H. hepatica*  Fonseca 1935, *H. achalai* Alzuet & Mauri, 1987, and *H. aiuabensis*  Bassini Silva, Jacinavicius & Welbourn, 2021 (Oudemans 1910, Sambon 1928, Fonseca 1935, Mendoza-Roldan et al. 2020, Bassini-Silva et al. 2021). However, the first four species were insufficiently described and the precise localities are unknown.

The Caatinga is the only biome unique to Brazil representing 11% of the Brazilian territory and is distributed throughout the northeast region. This biome is characterized by semiarid to arid climates with long dry seasons, irregular rainfall and high annual temperatures, and primarily composed of dry forest and shrubland. Unfortunately, it is one of the least protected and studied biomes in Brazil (Camardelli and Napoli 2012, Oliveira Arruda et al. 2024). Among the amphibian’s community at this biome, the Miranda’s white-lipped frog *Leptodactylus macrosternum* Miranda-Ribeiro, 1926 (Anura: Leptodactylidae) is an important component, a species with a wide distribution in South America and with the conservation status of least concern according with the red list of Ceará, Brazil, and the IUCN (Frost 2024, Oliveira Arruda et al. 2024). Here we describe a new chigger species of the genus *Hannemania* parasitic on *L. macrosternum* in the Caatinga, Brazil.

## Materials and Methods

We collected subdermal mites of the genus *Hannemania* on three specimens of *Leptodactylus macrosternum* during the wet season (May 2018) (*n* = 2) and dry season (September 2018) (*n* = 1). The study was conducted in the municipality of Petrolina, the Sub-middle São Francisco River region, state of Pernambuco, northeastern Brazil. This study is part of a greater research project about the parasitic mites on Brazilian herpetozoa and was performed under the authorization of the Instituto Chico Mendes de Conservação da Biodiversidade (ICMBio) (SISBIO/ICMBio #62680-1) and the Ethics Committee for the Use of Animals (CEUA) of the Universidade Federal do Vale do São Francisco Animal (UNIVASF #0001/221018).

The skin of the anurans was inspected under a stereomicroscope to collect mites, which were removed using a 26G ½ needle to cut the epidermis. The specimens were counted and preserved in 70% ethanol. Selected mites per each sample were separated to do semipermanent microscope slides using Hoyer’s medium as preserver following the method by Walter and Krantz (2009). Type specimens are deposited in the Acarological Collection of the Instituto Butantan (IBSP), São Paulo, Brazil, and voucher specimens also in IBSP and in the Colección Nacional de Ácaros (CNAC) of the Instituto de Biología, Universidad Nacional Autónoma de México, Mexico City, México, under the material transfer agreement (MTA/TTM No. 001/2024).

Mounted specimens were identified following the keys to the genera of chiggers of the Western Hemisphere (Brennan and Goff 1977), and posteriorly reviewing the original descriptions of similar species of the genus *Hannemania* (*ie*, Lahille 1927, Bassini-Silva et al. 2021). The mites were observed with a differential interference contrast and phase-contrast microscope (Nikon, Optiphot-2), and illustrations were made with a camera lucida adapted to it. Measurements were taken with an ocular micrometer and are in micrometers (μm). Selected mites were processed to take microphotographs in a scanning electronic microscopy (Hitachi, SU1510) following the protocol of Alberti and Nuzzaci (1996). Figures were ensembled using the GNU Image Manipulation Program (GIMP ver. 2.10.36) (The GIMP team 2023).

Palpal and leg setal nomenclature follows Grandjean’s system (1944, 1946) and idiosomal chaetotaxy follows Grandjean (1939) as it was implemented by Kethley (1990). Additional nomenclature, abbreviations and symbols follow Goff et al. (1982) and Stekolnikov (2013). The morphological traits mentioned in description, figures and measurements correspond to: (1) gnathosoma, *bs* = subcapitular seta (= gnathobasal seta); *cs* = adoral seta (= galeala); odo = odontus (= palpal claw); fPp = palpal setal formula, an expression of the setation of palpal femur/genu/tibia, these setae can be branched (= B), nude (= N) or with one branch or cilium (b). (2) For idiosoma, AA = distance between the bases of *vi* setae; AW = distance between the bases of *ve* setae; PW = distance between the bases of *sce* setae; ASB = distance from the trichobothria (*sci*) to extreme anterior margin of prodorsal sclerite; PSB = distance from bases of trichobothria *sci* to extreme posterior margin of prodorsal sclerite; SD = (ASB + PSB); AP = distance between the bases of *ve* and *sce*; OP = ocular plate; *vi* = internal vertical setae; *ve* = external vertical setae; *sci* = internal scapular setae, trichobothria; *sce* = external scapular setae; C, D, E, and F rows of dorsal idiosomal setae = idiosomal segments C, D, E and F, respectively; *3a* = posterior sternal setae (between coxae III); D_min_ = minimum length of dorsal opisthosomal setae; D_max_ = maximum length of dorsal opisthosomal setae; V_min_ = minimum length of ventral idiosomal setae; V_max_ = maximum length of ventral idiosomal setae. fSt = sternal setal formula, including numbers of anterior (between coxae I) and posterior sternal setae. (3) For legs: *1a* = proximal seta on coxa I; *1 b* = distal seta on coxa I; *2 b* = seta on coxa II; *3 b* = seta on coxa III; fCx = coxal setation formula, including numbers of setae on leg coxae I–III; σ I, σ II, and σ III = solenidia on genua I–III; nonspecialized setae on legs are branched (B); κ Ge I = microseta on genu I; κ Ti I = microseta on tibia I; φ′ I and φ″ I = distal and proximal solenidia on tibia I, respectively; ω I and ω II = solenidia on tarsi I and II, respectively; ε = famulus on tarsi I and II; ζ′ I = dorsal eupathidium on tarsus I; ζ″ I = subterminal eupathidium on tarsi I and II; z = companion seta on tarsus I; φ′ II and φ″ II = distal and proximal solenidion on tibiae II, respectively; ζ″ II = subterminal eupathidium on tarsus II; φ III = solenidion on tibia III. SIF = synthetic identification formula, that include selected characters of the palpus and legs, including the setation of the palpal tarsus, condition of the adoral seta, number of prongs on palpal claw, number of solenidia on genua I–III respectively, number of solenidia on tibia III, number of mastitarsalae III, number of mastitibialae III, number of mastigenualae III or presence of more than one genuala III, and number of mastifemoralae III.

In the description, the acronym of the acarological collection where the types and voucher specimens are deposited is given in square brackets, *ie* [IBSP] or [CNAC], while the collector numbers are in parentheses.

## Results

**Family Leeuwenhoekiidae Womersley, 1944**

**Genus *Hannemania* Oudemans, 1911**

Type species: *Heterothrombidium hylodeus*  Oudemans, 1910, by original designation.

**Fig. 1.**
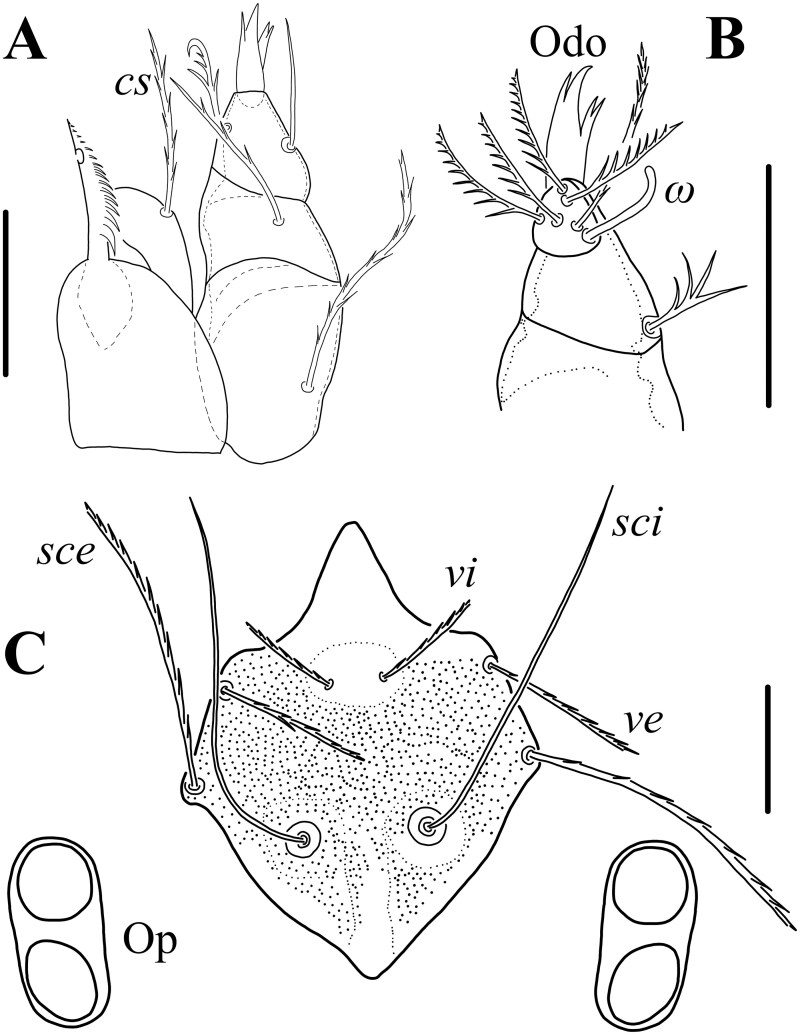
*Hannemania caatingensis* **sp. nov**. (holotype, larva): A) dorsal gnathosoma. B) ventral palp. C) prodorsal sclerite. Symbols: *cs* = adoral seta, *sce* = external scapular setae, *sci* = internal scapular setae, Odo = odontus, Op = ocular plate, *ve* = external vertical setae, *vi* = internal vertical setae and *ω* = solenidion. Scale bars: 25 μm.

**Fig. 2.**
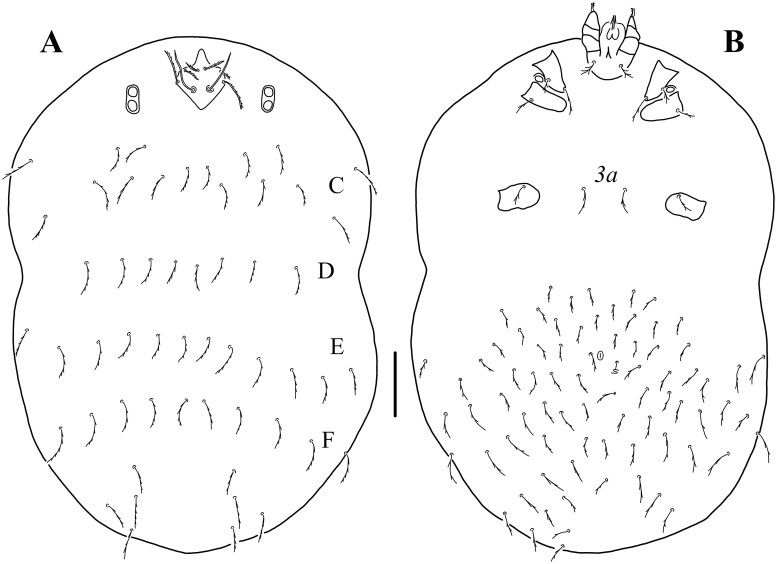
*Hannemania caatingensis* **sp. nov**. (holotype, larva): A) dorsal idiosoma. B) ventral idiosoma. Symbols: C–F rows of dorsal idiosomal setae = idiosomal segments C–F, respectively, *3a* = posterior sternal setae. Scale bar: 100 μm.

**Fig. 3.**
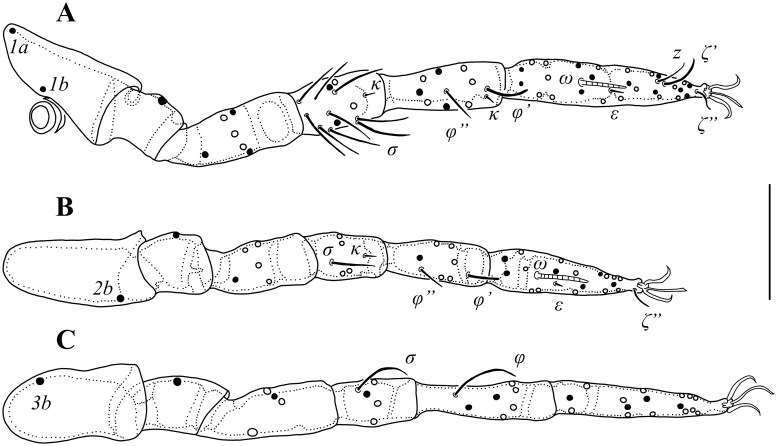
*Hannemania caatingensis* **sp. nov**. (holotype, larva): A) leg I. B) leg II. C) leg III. Symbols: *1a* = proximal seta on coxa I, *1 b* = distal seta on coxa I, *2 b* = seta on coxa II, *3 b* = seta on coxa III, *σ* = solenidia on genua I–III, *κ* = microseta on genua I–II and tibia I, *φ*, *φ’*, *φ”* = solenidia on tibiae I–III, *ω* = solenidia on tarsi I–II, *ε* = famulus on tarsi I–II, *z* = companion seta on tarsus I, ζ′ = dorsal eupathidium on tarsus I; ζ″ = subterminal eupathidium on tarsi I–II. Solid circles = ventral setae, open circles = dorsal setae. Scale bar: 50 μm.

**Fig. 4.**
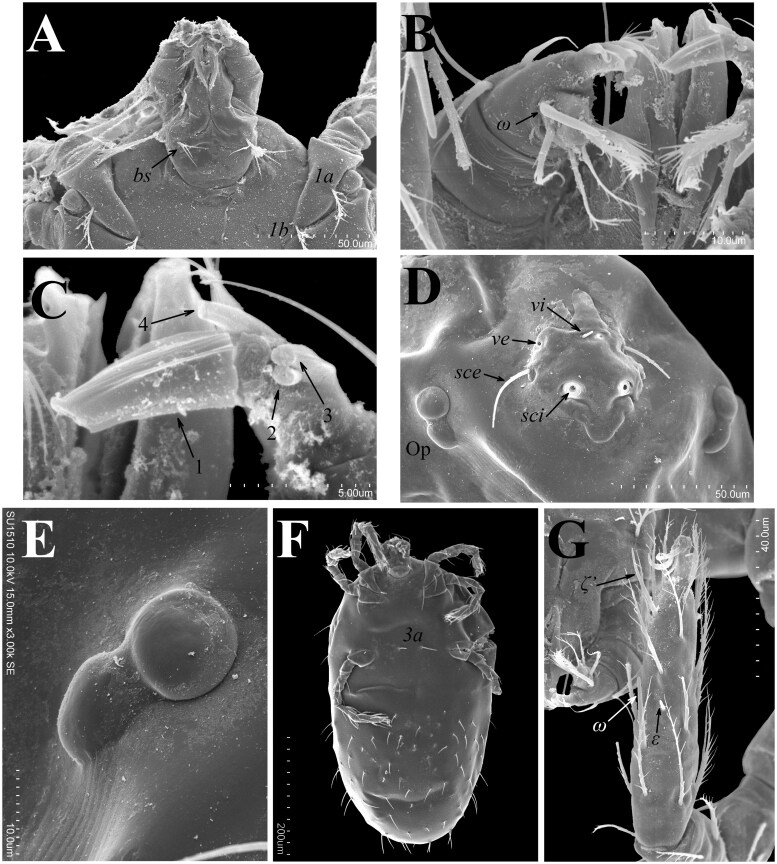
*Hannemania caatingensis* **sp. nov**. (paratype, larva), SEM micrographs: A) ventral aspect of gnathosoma. B) palpal tarsus. C) odontus. D) prodorsal sclerite and lateral ocular plates. E) ocular plate. F) ventral aspect of idiosoma. G) dorsolateral aspect of tarsus I. Symbols: *bs* = subcapitular seta, *1a* = proximal seta on coxa I, *1 b* = distal seta on coxa I, *3a* = posterior sternal setae, *ω* = solenidia on tarsus I and palpal tarsus, *vi* = internal vertical setae, *ve* = external vertical setae, *sci* = base (bothridium) of the internal scapular setae; *sce* = external scapular setae, Op = ocular plate, ζ′ = dorsal eupathidium on tarsus I; *ε* = famulus on tarsus I. Arrows and numbers in C indicate the prongs of odontus.

***Hannemania caatingensis* Paredes-León, Felix-Nascimento & Oliveira sp. nov. (** **Figs 1–4** **)**

(urn: lsid: zoobank.org: act: AEE01B52-DBC9-4D3B-8C6D-4B4BE344F3CC).

### Diagnosis

Larva with fPp: B/B/BNB/5Bω, odontus tetrafurcate, setae *cs* branched; two pairs of eyes located in ocular plates, trichobothria *sci* nude and flagelliform, relative length of scutal setae: *vi* < *ve* < *sce* < *sci*, setae *sce* relatively short (52–67 μm); dorsal hysterosoma with 54–56 setae: C row with 14 setae, D row with ten setae, E row with 12 setae, F row with ten setae, and eight or ten unordered setae posterior to F row and probably corresponding to an unrecognizable H row; 75–78 ventral opisthosomal setae; coxae III with *3 b*, seta *z* nude, 8–13 σ I and 1 σ II–III. SIF = 5B-B-4-(8-13)111.0000, fCx = 2.1.1., fSt = 0.2.

Larvae (holotype and 14 paratypes). *Gnathosoma*: palpal femur with branched and long (almost reaching tip of palpal tibia) dorsal seta, palpal genu with branched dorsal seta, shorter than that on femur, palpal tibia with one branched dorsal seta, one nude lateral seta and one branched ventral seta. Palpal tarsus with five branched setae and tarsala (ω); odontus tetrafurcate; cheliceral blade with row of 14–15 ventral distal teeth and a hook-shaped (like a bottle opener) dorsal tooth; setae *bs* and *cs* branched (Figs 1A and B and 4A–C).

*Idiosoma*: Ovoid, clearly longer than wide, maximum wide at level of C row. *Dorsum*: eyes 2/2 each pair on an ocular plate located on the sides of prodorsal sclerite (Figs 1C and 4E); prodorsal sclerite pentagonal-shape, with nasus, punctuated, with a pair of nude flagelliform trichobothria (*sci*) and six branched setae; *vi* < *ve* < *sce* < *sci* (Figs 1C and 4D); 54–56 dorsal setae apart from that on prodorsal sclerite: C row with 14 setae, D row with ten setae, E row with 12 setae, F row with ten setae, and eight or ten unordered setae posterior to F row and probably corresponding to an unrecognizable H row (Fig. 2A). *Venter*: one pair of sternal setae (*3a*) between coxae III and 75–78 opisthosomal setae; anus located at middle opisthosoma (Figs 2B and 4F; Table 1).

**Table 1.** Standard measurements of gnathosomal and idiosomal characters of the Holotype larva and 14 paratypes larvae of *Hannemania caatingensis*  **sp. nov.** (*n* = 15), except where noted.

Character	Range	Mean	Holotype
Gnathosoma length	85–115	100	115
Gnathosoma width	50–65	55	65
Idiosoma length	420–850	660	760
Idiosoma width (at level of coxae II)	245–580	415	460
Prodorsal sclerite width	62–80	69	75
V_min_	22–32	27	31
V_max_	41–58	46	58
D_min_	30–45	37	45
D_max_	43–55	47	55
OP length	35–40	37	39
OP width	18–22	20	19
SD	72–88 (*n* = 13)	76	87
SB	21–29	23	26
AA	7–11 (*n* = 14)	10	11
AW	43–57	47	54
PW	55–73	61	70
ASB	49–60 (*n* = 13)	52	60
PSB	23–31 (*n* = 13)	25	27
AP	13–20 (*n* = 14)	16	20
*vi*	20–28 (*n* = 12)	25	27
*ve*	28–35	32	35
*sce*	52–67	58	65
*sci*	75–85 (*n* = 13)	78	80

*Legs*: all six segmented and ending in a pair of claws and claw-like empodium, without onychotriches; tarsi I–II with apparent division into two non-articulating podomeres; *Leg I*: with two (*1a* and *1 b*) branched (2B) coxal setae, trochanter 1B, femur 6B, genu 4B, 8–13 σ and distal κ, tibia 8B, 2 φ (φ′ and φ″) and distal κ, tarsus 26B, ω, ε, ζ′, z (nude), and ζ″ (Figs 3A and 4G); *Leg II*: coxa with one (*2 b*) branched seta (1B), trochanter 1B, femur 5B, genu 4B, 1 σ and distal κ, tibia 6B and 2 φ (φ’ and φ″), tarsus 18B, ω, ε, and a ζ″ (Fig. 3B); *Leg III*: coxa with one (*3 b*) branched seta (1B), trochanter 1B, femur 4B, genu 4B and 1 σ, tibia 6B and 1 φ, tarsus 16B (Fig. 3C; Table 2).

**Table 2.** Standard measurements of leg characters of the Holotype larva and 14 paratypes larvae of *Hannemania caatingensis* **sp. nov.** (*n* = 15), except where noted.

Character	Range	Mean	Holotype
Leg I length (from coxa to tarsus)	250–330	300	320
Tarsus I length	73–86	76	85
σ on genu I	22–25 (*n* = 14)	23	25
κ on genu I	4–5	4	4
φ’ on tibia I	19–22 (*n* = 14)	20	22
φ” on tibia I	17–19 (*n* = 14)	17	19
κ on tibia I	4–6 (*n* = 14)	5	4
ε on tarsus I	6 (*n* = 14)	6	6
ζ’ on tarsus I	22–24 (*n* = 14)	23	23
z on tarsus I	17–18 (*n* = 14)	18	17
ζ” on tarsus I	11–13 (*n* = 14)	13	11
ω on tarsus I	21–24	23	22
Leg II length (from coxa to tarsus)	210–290	257	290
σ on genu II	23–26 (*n* = 14)	24	25
κ on genu II	5–6 (*n* = 14)	6	6
φ’ on tibia II	15–20 (*n* = 14)	18	15
φ” on tibia II	15–16 (*n* = 14)	16	16
ε on tarsus II	4 (*n* = 14)	4	4
ζ” on tarsus II	11–13 (*n* = 14)	12	11
ω on tarsus II	24–27	25	25
Leg III length (from coxa to tarsus)	250–325	285	325
σ on genu III	23–29 (*n* = 14)	25	29
φ on tibia III	24–28 (*n* = 14)	25	28

### Remarks

We observed variations in the number of setae as follows: (1) The number of σ I (8–13) was variable among specimens and even in the legs I in the same specimen, 8/8—four paratypes, 8/9—holotype and two paratypes, 9/9—one paratype, 9/10—one paratype, 9/11—two paratypes, 10/10—two paratypes, 10/12—one paratype and 10/13—one paratype; (2) the lateral setae on palpal tibia have one or two branches (= b) in two paratypes instead of being nude (N); (3) one paratype has three *vi* setae instead of two.

### Type Series

HOLOTYPE larva and 1 PARATYPE larva, ex *Leptodactylus macrosternum*, Conventional area, municipality of Petrolina, state of Pernambuco, Brazil, 09.334633° S, 40.586458° W, 28-V-2018, coll. G. Felix-Nascimento (AAC15) [IBSP24190]. 7 PARATYPE larvae , Organic area, municipality of Petrolina, state of Pernambuco, Brazil, 09.004958° S, 40.291894° W, 23-V-2018, coll. G. Felix-Nascimento (AA013) [IBSP24191]. 6 PARATYPE, larvae, Caatinga *senso stricto* area, municipality of Petrolina, state of Pernambuco, Brazil, 09.126550° S, 40.358553° W, 30-IX-2018, coll. G. Felix-Nascimento (ACC21) [IBSP24192].

### Type Depository

Holotype and 14 paratypes will be deposited at the Acarological Collection of the Instituto Butantan [IBSP], São Paulo, Brazil.

### Additional Material Examined

One larva [CNAC012389], same data as Holotype except (AAC15.4); 15 larvae preserved in ethanol [IBSP24191] and 20 larvae preserved in ethanol [CNAC012381], same data as Paratypes AA013, except (AA013.9); 6 larvae preserved in ethanol [IBSP24192] and 6 larvae preserved in ethanol [CNAC012392], same data as Paratypes ACC21, except (ACC21.8).

### Distribution

Known only in the municipality of Petrolina, Brazil.

### Etymology

The specific name, *caatingensis*, is formed by adding -ensis to the biome name Caatinga of Brazil where the type locality is situated, to produce a Latinized adjectival name meaning “from Caatinga.”

### Differential Diagnosis

*Hannemania caatingensis* sp. nov. differs from all other *Hannemania* by having the odontus with more prongs (four) than the other species, except for *H. argentina*  Lahille, 1927 that has one to five prongs (Lahille 1927). However, *Hannemania caatingensis* sp. nov. can be separated from *H. argentina* by its fPp (B/B/BNB) and by having *bs* seta branched, whereas that in *H. argentina* the fPp is N/B/BBB and *bs* is nude.

It should be noted that in Table 3 of Silva-de la Fuente et al. (2016) the number of prongs in odontus for *H. argentina* and *H. samboni* Ewing 1931 is misinterpreted: (1) in *H. argentina* odontus has one to five prongs (see Lahille 1927: 9) instead of unknown, and (2) in *H. samboni* odontus is trifurcate (see Sambon 1928: 131) instead of having one to five prongs.

## Discussion

*Hannemania caatingensis* sp. nov. is the 29th species of the genus *Hannemania* and the seventh species of this genus recorded in Brazil. The host of *H. caatingensis* sp. nov., the Miranda’s white-lipped frog, is also one of the hosts of *H. aiuabensis* in Aiuaba municipality, Ceará, Brazil. However, these chigger species are easily separated by the following characteristics: (1) the number of branched setae on tarsi I (26 in *H. caatingensis* sp. nov. and 25 in *H. aiuabensis*); (2) the number of dorsal and ventral idiosomal setae (54–56 and 75–78 in *H. caatingensis* sp. nov. and 44–46 and 39–43 in *H. aiuabensis*); (3) the ocular plate (present in *H. caatingensis* sp. nov. and absent in *H. aiuabensis*); (4) the number of odontus prongs (odontus is tetrafurcate in *H. caatingensis* sp. nov. and trifurcate in *H. aiuabensis*); (5) the fPp (B/B/BNB in *H. caatingensis* sp. nov. and B/N/NNB in *H. aiuabensis*); and (6) the length of *sce* seta (52–67 in *H. caatingensis* sp. nov. and 29–31 in *H. aiuabensis*) (Bassini-Silva et al. 2021). All these morphological differences show that *H. caatingensis* sp. nov. and *H. aiuabensis* are not conspecific, despite parasitizing the same frog species. The localities of both chigger species are separated by a distance of at least 240 km in a straight line.

The genus *Hannemania* seems more diverse as more hosts are analysed. However, due to the brief description of most species, the genus needs a systematic review that includes an effort to analyse type specimens and obtain fresh material from type localities to explore molecular characters to separate species unequivocally.

## Nomenclature

This paper and the nomenclatural act it contains have been registered in Zoobank (www.zoobank.org), the official register of the International Commission on Zoological Nomenclature. The LSID (Life Science Identifier) number of the publication is: urn:lsid:zoobank.org:pub:3B8E096C-65AA-47FA-9E61-A349864D0371.
